# Association of gut microbiota dysbiosis with non-alcoholic fatty liver disease: A cross-sectional study

**DOI:** 10.6026/973206300212477

**Published:** 2025-08-31

**Authors:** Lalan Pratap Singh, Ruchi Agrawal, Preeti Nigotia, Ramesh Agrawal

**Affiliations:** 1 Department of General Medicine, Government Medical College, Satna, Madhya Pradesh, India; 2 Department of Microbiology, Bundelkhand Medical College, Sagar, Madhya Pradesh, India; 3 Department of General Medicine, SRVS Medical College, Shivpuri, Madhya Pradesh, India; 4 Department of Microbiology, Government Medical College, Santa, Madhya Pradesh, India

**Keywords:** NAFLD, gut microbiota, dysbiosis, liver disease, microbial diversity

## Abstract

The association between gut microbiota dysbiosis and non-alcoholic fatty liver disease (NAFLD) in 132 adults were diagnosed using ultrasonography. Stool samples were analyzed using 16S rRNA sequencing to assess microbiota composition. Patients with NAFLD showed a significant decrease in *Bacteroidetes* and an increase in *Firmicutes* and *Proteobacteria* compared to controls. Altered microbial diversity correlated with elevated liver enzymes and insulin resistance markers. Data shows that gut microbiota imbalance plays a contributory role in the pathogenesis of NAFLD.

## Background:

Non-alcoholic fatty liver disease (NAFLD) has emerged as the most prevalent chronic liver condition globally, affecting approximately 25-30% of the adult population [1]. It encompasses a spectrum ranging from simple hepatic steatosis to non-alcoholic steatohepatitis (NASH), which can progress to cirrhosis and hepatocellular carcinoma [2]. Perturbations in gut microbiota are associated with NAFLD, commonly reflected by a reduction in beneficial species and an increase in the pathogenic species [4]. The relationship between the gut microbiome and the risk of developing NAFLD was summarized as odds ratios (ORs) and 95% confidence intervals (95% CIs) [5]. Unlike other liver diseases, NAFLD occurs in individuals who consume little to no alcohol and it is closely associated with metabolic disorders such as obesity, type 2 diabetes mellitus and dyslipidemia and insulin resistance [3]. Recent advances in microbiome research have identified the gut-liver axis as a key player in liver health and disease [6]. The gut microbiota, comprising trillions of microorganisms, plays a crucial role in maintaining intestinal homeostasis and metabolic balance [7]. Dysbiosis a state of microbial imbalance has been implicated in the development and progression of NAFLD through mechanisms such as increased gut permeability, endotoxemia, inflammation and altered short-chain fatty acid production [8]. Despite growing evidence, the precise microbial patterns associated with NAFLD remain underexplored in many populations [9]. Therefore, it is of interest to investigate the association between gut microbiota dysbiosis and NAFLD in adults, with the objective of identifying microbial signatures that could serve as potential biomarkers or therapeutic targets in NAFLD management.

## Materials and Methods:

This cross-sectional study was conducted at a tertiary care hospital over a period of 12 months and included 132 adult participants aged 25 to 60 years. Participants were recruited from the outpatient department, with 72 patients diagnosed with non-alcoholic fatty liver disease (NAFLD) based on abdominal ultrasonography and 60 age- and sex-matched controls without NAFLD. Individuals with a history of alcohol consumption, viral hepatitis, autoimmune liver disease, recent antibiotic or probiotic use (within the past 3 months), gastrointestinal surgery, or other chronic systemic illnesses were excluded. After obtaining informed consent, clinical and anthropometric data including BMI, waist circumference, and blood pressure were recorded. Fasting blood samples were collected to assess liver function tests, fasting glucose, insulin levels, and lipid profiles. Homeostatic Model Assessment of Insulin Resistance (HOMA-IR) was calculated. Stool samples were collected from all participants and immediately stored at -80°C. Microbial DNA was extracted using a standardized commercial kit, and the V3-V4 region of the 16S rRNA gene was amplified and sequenced using Illumina MiSeq technology. Bioinformatic analysis was performed to determine microbial diversity and relative abundance at phylum and genus levels. Statistical analysis was carried out using SPSS version 26.0. Continuous variables were compared using t-tests or Mann-Whitney U tests, while categorical variables were compared using chi-square tests. A p-value < 0.05 was considered statistically significant.

## Results:

A total of 132 participants were included, of which 72 had NAFLD and 60 served as healthy controls. The NAFLD group showed significant differences in metabolic parameters and gut microbiota composition compared to the control group. The following tables present the demographic, biochemical, and microbial diversity findings with relevant interpretations. Table 1 (see PDF) presents the baseline demographic and anthropometric characteristics of the participants. Individuals with NAFLD had significantly higher BMI, waist circumference, and systolic and diastolic blood pressure compared to controls. These findings highlight a strong association between NAFLD and metabolic risk factors, even though age and sex distribution were similar between the groups. Table 2 (see PDF) outlines key biochemical and metabolic parameters. NAFLD patients showed significantly elevated levels of ALT and AST, along with higher fasting glucose, insulin, and HOMA-IR scores, confirming the presence of insulin resistance and hepatic dysfunction within this group. Table 3 (see PDF) compares the relative abundance of major gut microbial phyla. NAFLD patients exhibited a markedly increased proportion of *Firmicutes* and *Proteobacteria* and a reduced abundance of *Bacteroidetes*. This shift indicates a dysbiotic gut environment commonly associated with metabolic disorders. Table 4 (see PDF) reports gut microbial diversity indices. The Shannon and Simpson indices, along with Chao1 richness, were all significantly lower in the NAFLD group, suggesting both reduced microbial richness and evenness hallmarks of dysbiosis. Table 5 (see PDF) details the abundance of selected bacterial genera. Pro-inflammatory and endotoxin-producing genera like *Escherichia*, *Enterococcus*, and *Clostridium* were significantly elevated in NAFLD participants, whereas beneficial genera such as *Bacteroides* and *Prevotella* were notably reduced, reflecting an inflammatory and metabolically harmful microbiota. Table 6 (see PDF) shows the correlation between microbial phyla and HOMA-IR scores. *Firmicutes* and *Proteobacteria* had strong positive correlations with insulin resistance, while *Bacteroidetes* showed a negative correlation. This suggests a mechanistic link between dysbiosis and metabolic dysfunction in NAFLD. Table 7 (see PDF) stratifies gut microbial profiles by NAFLD severity. As steatosis severity increased from mild to severe, there was a progressive rise in *Firmicutes* and a decline in *Bacteroidetes* and microbial diversity, implying a gradient effect of dysbiosis on disease progression. Table 8 (see PDF) presents multivariate logistic regression analysis for predictors of NAFLD. After adjusting for age, BMI, and lipid profile, both *Firmicutes* abundance and HOMA-IR emerged as independent predictors of NAFLD, suggesting their strong pathophysiological role. Table 9 (see PDF) provides ROC curve analysis of microbial indices for NAFLD detection. The Shannon index and *Firmicutes* proportion showed good discriminatory power (AUC > 0.79), suggesting their potential utility as non-invasive microbial biomarkers for NAFLD screening. Table 10 (see PDF) compares liver enzyme levels between patients with high and low *Firmicutes*:*Bacteroidetes* (F:B) ratios. Those with elevated F:B ratios had significantly higher ALT and AST levels, reinforcing the microbial-hepatic axis and suggesting that microbial imbalance directly influences liver injury.

## Discussion:

This cross-sectional study highlights a significant association between gut microbiota dysbiosis and non-alcoholic fatty liver disease (NAFLD). Participants with NAFLD exhibited marked alterations in microbial composition, including a higher abundance of *Firmicutes* and *Proteobacteria* and a reduction in *Bacteroidetes*, along with lower microbial diversity indices [10]. These findings are consistent with emerging literature suggesting that gut microbial imbalance may contribute to the pathogenesis of NAFLD through mechanisms involving increased intestinal permeability, endotoxemia and systemic inflammation [11]. The elevated *Firmicutes*: *Bacteroidetes* ratio observed in NAFLD subjects aligns with patterns seen in obesity and insulin resistance, further emphasizing the role of the gut-liver axis [12]. Moreover, the positive correlation between *Firmicutes* abundance and HOMA-IR, as well as the inverse relationship with microbial diversity, suggests that dysbiosis may influence hepatic lipid accumulation and insulin signaling pathways [13]. Increased levels of *Escherichia*, *Enterococcus* and *Clostridium* known to produce endotoxins and promote inflammation were also seen in the NAFLD group, reinforcing the potential role of microbial endotoxins in liver injury [14]. Importantly, the degree of dysbiosis appeared to worsen with increasing severity of steatosis, indicating a possible dose-response relationship [15]. Our findings also highlight that microbial markers, particularly the Shannon diversity index and relative abundance of *Firmicutes*, have good diagnostic potential, as evidenced by ROC curve analysis [16]. These microbial patterns could serve as non-invasive biomarkers for early detection and risk stratification of NAFLD [17]. However, this study is limited by its cross-sectional design, which precludes causal inferences. Longitudinal studies and interventional trials are needed to explore whether modulation of gut microbiota can prevent or reverse NAFLD. Overall, this study reinforces the relevance of the gut microbiome in NAFLD and supports its potential as a therapeutic target.

## Conclusion:

A significant association between gut microbiota dysbiosis and non-alcoholic fatty liver disease is shown. Altered microbial diversity and increased abundance of pro-inflammatory taxa correlate with hepatic dysfunction and insulin resistance. Targeting gut microbiota may offer promising strategies for early diagnosis and management of NAFLD.
